# Multifunctional Role of Cytokinin in Horticultural Crops

**DOI:** 10.3390/ijms26031037

**Published:** 2025-01-25

**Authors:** Shahid Hussain, Jingjing Chang, Jing Li, Lei Chen, Sheraz Ahmad, Zhao Song, Baige Zhang, Xiao Chen

**Affiliations:** 1 Key Laboratory for New Technology Research of Vegetable, Vegetable Research Institute, Guangdong Academy of Agricultural Science, Guangzhou 510640, China; hussainshahid@gdaas.cn (S.H.); jingkyd@163.com (J.C.); ljing1@webmail.hzau.edu.cn (J.L.); chenlei1@gdaas.cn (L.C.); songzhao@gdaas.cn (Z.S.); plantgroup@126.com (B.Z.); 2 Guangdong Provincial Key Lab of Agro-Animal Genomics and Molecular Breeding, College of Animal Science, South China Agricultural University, Guangzhou 510642, China; sherazahmad@scau.edu.cn

**Keywords:** cytokinin, flowering, fruit setting, stress biology, physiology

## Abstract

Cytokinins (CKs) are a class of phytohormones identified in the early 1960s and are mainly responsible for stimulating cell division. Following the discovery, research to help understand the pluralistic roles of CKs in plant growth and stress biology increased. With their fascinating ability, CKs serve as an important element in regulating the defense–growth trade-off. Herein, we demonstrate how the CK fine-tuning the organogenesis of different parts of horticultural plants is discussed. CK’s role in tailoring reproductive biology (flowering, sex differentiation, fruit set, and fruit attributes) has been presented. An extensive explanation of the CK-mediated response of horticultural crops to abiotic (temperature, drought, and salinity) and biotic stresses (fungal, bacterial, and nematodes) is provided. Finally, we posit the unexplored roles of CKs and highlight the research gaps worth addressing.

## 1. Introduction

Cytokinins (CKs) were first identified as promoting de novo organ development in a tissue culture experiment [1]. CKs boost cytokinesis, which is crucial to the structural makeup of plants [2]. In recent decades, CKs have been implicated in root-to-reproductive-biology development [2,3]. Multiple studies have identified crucial genes involved in the biosynthesis, transportation, and signaling of CK [4].

CK is generally regarded as a growth hormone, finely tweaking the different growth phases of plants. For instance, root growth mainly depends on CK basal biosynthesis. CK signaling regulates the longitudinal zonation in the root meristem by controlling cell differentiation. CKs control radial patterning of root vasculature by promoting protophloem cell identity and spatially inhibiting protoxylem formation [5]. The pivotal role of CKs in leaf development has been extensively discussed by [6]. The promotion of cell division and expansion during the proliferative stages of leaf cell development was investigated in CK-treated leaves [6]. CK is an essential constituent in modulating plant architecture. An excellent example of CK mediating plant architecture was documented in the dwarf cucumber. The *compact* (*cp*) mutant in cucumber encoding *Cytokinin oxidase*/*dehydrogenase 1* displayed an extremely dwarf phenotype [7]. CK’s role in flower and fruit induction is evident from several reports [3,8,9]. In particular, CK is used very often in producing parthenocarpic fruit [3]. Besides that, CK’s involvement in regulating plant response to abiotic and biotic stresses speaks volumes about its versatile nature. An induction of the CK content has been observed in cucumber plants subjected to heat stress [10]. Transgenic creeping bentgrass (*Agrostis stolonifera*) plants harboring the *isopentenyltransferase* (*ipt*), a CK biosynthesis gene, displayed better resistance to drought stress [11]. CK’s interaction with salicylic acid (SA) and ethylene (ETH) augmented the immune response of tomato plants to *Botrytis cinerea* (*B. cinerea*), *Xanthomonas campestris pv. vesicatoria* (*Xcv*) and *Pseudomonas syringae pv. tomato* (*Pst*) [12,13]. More recently, CK’s role has been investigated in postharvest biology and technology. CK delayed the ripening and senescence of mango fruit by inhibiting the biosynthesis of ETH and lipid catabolism [14]. Although the review of [15] has previously presented the role of CK in horticultural crops, our article updated the information.

In our article, we extensively discuss the molecular regulatory role of CKs in horticultural crops. Firstly, we briefly explain CK biosynthesis, transport, and signaling. Following that, CK-mediated plant growth activities are presented in detail. The CK and its potential role in regulating the immune response of plants to various abiotic and biotic stresses are discussed. Critical discussion, research gaps, and future perspectives are simplified to help early-stage researchers comprehend CKs’ multifunctional role. To the best of our knowledge, our article will be the first to explain the molecular biology of cytokinin in horticultural crops. We believe our manuscript will enhance the understanding of these essential molecules and help in future breeding programs.

## 2. Cytokinin Biosynthesis, Transport, and Signaling

In addition to inactive precursors like tZ-riboside (tZR), and iP-riboside (iPR), plants produce four other forms of active CKs: isopentenyltransferase (IPT), tZ, cis-zeatin (cZ), and dihydrozeatin (DZ) [3]. An IPT catalyzes the additional prenyl group from dimethylallyl diphosphate (DMAPP) to the N6 position of AMP, ADP, or ATP at the start of CK production [2]. The tRNA-IPT s, which synthesize cZ-type CK, are poorly known and assumed to be housekeepers throughout plant growth and development. While ATP/ADP-IPTs are expressed tissue-specifically and swiftly downregulated by abiotic stressors, tRNA-IPTs are expressed constitutively throughout the plant [16]. The final product, isopentenyl adenosine 5′-phosphates (iP nucleotides), is further converted to tZ derivatives by cytochrome P450 trans-hydroxylases (CYP735A1 and CYP735A2) [17]. LOG (LONELY GUY) encodes a novel CK-activating enzyme that works in the final step of bioactive CK synthesis. The LOG enzyme directly transforms inactive CK nucleotides into their physiologically active free-base forms (iP and tZ) through CK-specific phosphoribohydrolase activity [18,19]. The CK Oxidase/Dehydrogenase (CKXs) cleave both iP and tZ, but dihydrozeatin and synthetic CKs such as 6-benzylaminopurine (6-BAP) and kinetin are not broken down by CKXs [20,21]. Biotic and abiotic variables and endogenous developmental signals control the production and degradation of CK [22].

CK are generated in roots and shoots and can move between roots and shoots and between adjacent cells [2]. For CK translocation, three different kinds of membrane transporters have been identified. The CK nucleobases and nucleosides are transported via the equilibrative nucleoside transporters (ENTs) and purine permeases (PUPs), which act as influx transporters. The ATP-binding cassette transporter subfamily G14 (ABCG14) in *Arabidopsis* functions as an efflux pump, enabling the root-born CK to translocate across extended distances [23].

Transduction of the CK signal occurs via a two-component system (TCS) to the target genes. The CK signaling pathway in *Arabidopsis* involves three distinct sets of proteins: type-B response regulators (type-B ARRs), histidine kinases (AHKs), and histidine-containing phospho-transfer proteins (AHPs) [24]. Autophosphorylation of AHKs occurs in response to CK. The phosphoryl group is transported to the type-B ARRs via AHPs. Subsequently, target DNA is bound by phosphorylated type-B ARRs, which promote the expression of genes that react to CKs. The *Arabidopsis* genome contains type-A ARR and type-B ARRs genes [25]. Similar to type-B ARRs, type-A ARRs possess a receiver domain for obtaining a phosphoryl group from AHPs but lack a DNA-binding domain (GARP domain). Therefore, type-A ARRs disrupt the CK signaling pathway by competing with type-B ARRs for the phosphoryl group [25,26]. A thorough explanation of the transport, signaling, and CK biosynthesis has been addressed by [27].

## 3. Cytokinin Is Instrumental in Developmental Biology

CK is a primary growth regulatory hormone, fine-tuning every plant developmental stage. Below, we discussed the instrumental role of CK in regulating the developmental biology of horticultural plants. Table 1 presents the various roles of CK and its related genes in the growth biology of horticultural crops.

### 3.1. Cytokinin’s Role in Regeneration

As sessile organisms, plants have evolved and perfected the skill of adaptability. The capacity to regenerate or substitute damaged tissues and structures is probably one of their most remarkable accomplishments [28]. The extraordinary plasticity of developmental and regenerative pathways is attributed to the restoration of cellular totipotency. According to the definitions provided by Gamborg and Phillips, regeneration can occur through organogenesis, which involves the formation of distinct organs (e.g., shoots or roots), or through somatic embryogenesis, characterized by the development of a bipolar structure that encompasses both shoot and root meristems, resembling the development of zygotic embryos [28,29]. The transcriptional activation of *WUSCHEL* (*WUS*) is regarded as a pivotal molecular event that initiates CK-induced shoot organogenesis. During the creation of SAM, *WUS* is believed to have delineated the organizing center (OC) [29,30]. Consequently, *WUS* expression is critical for the de novo formation of shoot stem cell niches, which produce signals that regulate the equilibrium between self-renewal processes and the generation of daughter cells capable of differentiating into new tissues, as well as the induction of stem cell identity in the cell population that overlays the OC. Meristem maintenance relies on a balance between stem cell division in the meristem core and differentiation at the periphery [30,31]. The overexpression of *WUS* distinctly results in ectopic shoot development. Given that *WUS* mutants exhibited no shoot regeneration in vitro, it is inferred that the activation of identity acquisition in shoot meristematic progenitor cells necessitates a specific threshold of *WUS* gene expression [31]. Research work on regeneration in horticultural plants has received attention recently. CK utilization in this regeneration work could be of great significance.

### 3.2. Cytokinin and Root Biology

Since CKs are widely recognized to impede root growth and development, some mutants that block their production have longer primary roots than wild-type examples [32]. Arbuscular mycorrhizal (AM) fungi and *Serendipita indica* are examples of root-associated endophytic fungi that can enhance root formation and plant development [33]. To monitor changes in biomass, root morphological characteristics, and CK concentrations in roots and trifoliate orange (*Poncirus trifoliata*) seedlings were inoculated with the AM fungus *Funneliformis mosseae* and *S. indica*. Shoot and root biomass, root total length, taproot length, average diameter, surface area, volume, and the number of lateral roots all improved after 20 weeks of inoculation with these fungi. Additionally, endophytic fungal inoculation significantly induced the concentration of tZ, dZ, and IPT in roots. These findings imply that improvements in plant development and root architecture brought about by root-associated endophytic fungus were linked to modifications in endogenous CK [33].

Adventitious roots (ARs) are important constituents of the root system, playing a crucial role in development and stress response. CKs are known as the negative regulator of AR formation in horticultural crops. The application of 6-benzyladenine (6-BA) to apple significantly suppressed ARs [34]. The CK signaling gene *MdRR12* was respectively induced and suppressed significantly after treatment of 6-BA and lovastatin (Lov), a synthetic cytokinin inhibitor. To confirm, transgenic plants carrying *35S::MdRR12* displayed increased sensitivity to exogenous CK and inhibited ARs phenotype [34]. Similarly, in tea (*Camellia sinensis* L.), auxin application promoted AR formation by suppressing the expression of CK signaling genes [35].

Despite the compelling role of CK in regulating root biology, the research body is scarce in addressing the molecular framework underlying these changes. For instance, the CK crosstalk with other root-associated hormones such as ethylene and auxin could be investigated to understand how these molecules architect the complex signaling pathway that governs root development.

### 3.3. Cytokinin Regulates Vegetative Growth

Leaf and stem are the primary photosynthetic organs, crucial for the growth and survival of plants [36]. CK maintains the development of shoot apical meristems, which supply stem cells to create leaf primordia during the first leaf formation stage [37]. Together, the actions of CK in different leaf regions, particularly the margins, determine the ultimate leaf shape. The quantity and dimensions of a leaf’s cells typically dictate its area. During leaf growth’s proliferation and expansion phases, CK stimulates cell division and cell expansion, respectively. Crop development is determined by the source and sink balance, primarily influenced by nitrogen (N) supply [38]. As signaling molecules, CKs are crucial for long-distance organ-to-organ communication in regulating systemic growth in response to N [39]. The response of potato plants to N revealed that mixed N (75%/25% nitrate/ammonium) improved leaf area, photosynthetic activity, and N metabolism compared to a solitary nitrate supply. Exogenously applied 6-BA increased photosynthesis and leaf area, with results comparable to those of mixed N [38]. Silencing the *SlIPT4* gene in tomatoes causes early leaf senescence [40]. SlymiR208, a recently identified microRNA in tomatoes, regulates the expression of *SlIPT2* and *SlIPT4* at the post-transcriptional level. SlymiR208 expression is highly expressed in aged leaves and transitions differently from its target transcripts [40]. Overexpressing SlymiR208 in tomato plants resulted in a considerable decrease in the amounts of endogenous CKs in leaves as well as the transcript levels of *SlIPT2* and *SlIPT4*. The overexpression of SlymiR208 led to premature leaf senescence, which was consistent with the phenotype observed in *SlIPT4* silenced lines [40]. Although many studies advocate the role of CK in leaf biology, the complex molecular mechanism of how these delicate processes take place is still unclear. It would be fascinating to investigate how CK changes the morphology and structure of leaves or how the CK biosynthesis gene influences the development of other plant parts. Leaf senescence and chlorophyll-degradation-related genes (*NAC*) [41] and hormones such as ethylene [42] in crosstalk with CK during leaf development would be of value to extend our understanding.

Shoot branching is essential to plant architecture. In many species, including the potato (*Solanum tuberosum* L.), sugars are crucial in causing shoot branching [43]. Feeding sucrose to disconnected stems increases the production of the vacuolar invertase (VInv) enzyme, which strengthens the sugar sink and the build-up of CK. In the absence of sucrose, CK-supplied detached stems stimulated bud development and VInv activity, whereas alternative results were observed when CK biosynthesis and signaling inhibitors were applied. In *vinv* mutants, bud outgrowth triggered by CK was inhibited. The findings imply that CK-mediated induction of VInv activity is partially responsible for sugar-induced lateral bud expansion [43]. Compact plant varieties suitable for high-density planting and once-over mechanical harvesting are recommended when producing cucumbers. There are now two cucumber mutants with dwarf phenotypes: *compact* (*cp*) and *compact-1* (*cp-1*). Due to their very small internodes, the *cp* and *cp-1* mutants have minimal to no usefulness in practical applications. The shorter internodes in the *cp* and *cp-1* mutants can be attributed to a decrease in the number of cells in the main stem and a lower presence of endogenous CK. The genes *CKX* and *Cullin 1* are responsible for the specific phenotypes observed in the *cp* and *cp-1* mutants, respectively [44]. *SlRR6*, a key CK signaling gene, regulates the intricate relationship between CK signaling and the GA and IAA networks that determine tomato plant height [45]. When overexpressed, it inhibits IAA synthesis and increases GA accumulation by acting downstream of the CK signaling transduction pathway. This results in cell division and expansion changes, which control plant height [45]. The *SlRR6* is impacted by feedback regulation; specifically, the expression of its mRNA is induced by GA and inhibited by IAA. It is worth mentioning that the gene *SlSAUR58*, which responds to IAA, directly interacts with the *SlRR6* protein. This interaction may help in the IAA-mediated negative regulation of SlRR6. Despite these latest findings, more investigation is necessary to complete the network and understand the puzzling signaling mechanism [45]. *MdIPT1* was significantly up-regulated during axillary bud development [46]. The *MdIPT1* promoter exhibited strong activity in various tissues and reacted to several hormone treatments. The multi-branching phenotype of the *MdIPT1*-overexpressing *Arabidopsis* was accompanied by a higher concentration of endogenous CK and a shift in the expression of genes involved in branching [46]. However, CK’s role in regulating branching and plant height is far from clear. For instance, the upregulation in *pIPT::GUS* promoter activity was observed in response to ABA, which is generally considered as stress hormone. How IAA and CK regulatory network works during vegetative growth in the absence of GA and other hormones also need further research.

### 3.4. Cytokinin in Flower Development

Plants respond to manipulative interventions such as girdling, trimming, and plant growth regulators that can result in blooming in non-inductive natural conditions [47]. A series of articles have discussed CK’s role in flower development.

#### 3.4.1. Floral Induction

The process via which stimuli from outside the shoot apex cause flower primordia to develop is known as floral induction. CK is pivotal in floral induction amongst various horticultural species. For example, potassium chlorate (KClO_3_) treatments were applied to longan trees (*Dimocarpus longan* Lour.) in non-conducive environmental circumstances, alone or in conjunction with shoot girdling or defoliation treatments [48]. Four weeks following treatment, treated trees displayed visible flower buds, whereas reference plants that were not treated remained vegetative. The transitory up-regulation of the flower-promoting gene *Dimocarpus longan FLOWERING LOCUS 1* (*DlFT1*) in mature leaves following KClO_3_ treatment correlates with the higher CK content in shoot apical tissues, which is most likely the result of de novo production and transport. Chemically inducing longan blooming appears to involve precise coordination of hormonal and genetic pathways, as evidenced by the distinct pattern of CK buildup and flowering gene expression [48]. The apple was treated with 6-BA at the key flower transition stage to evaluate the role of CK [49]. The 6-BA treatment significantly induced flowering in apples compared to controls. Post 6-BA treatment, increased accumulation of CK and expression of CK signaling genes (*AHK*, *AHP*, and *ARRs*) were observed. In 6-BA treated buds, the expression levels of genes involved in sucrose production and T6P metabolism, including *SUS*, *SPS*, *TPS*, and *TPP*, were greater. The T6P pathway is key in phase transition by repressing the expression of miR156 thus accelerate flowering [50]. It is important to know that miR156 is a key suppressor of flower induction and fruit development [51]. It implies that through controlling sink strength and sugar signaling, the 6-BA encourages apple floral induction. Furthermore, the 6-BA treatment had a substantial impact on genes in the GA, age, and photoperiod flowering pathways (*SOC1*, *SPL5*, *SPL6*, *SPL8*, *SPL14*, *SPL15*, *WOX1*, and *PIF*) [49]. Complementary to this, the *MdIPT1*-overexpressing plants displayed an early-flowering phenotype, with increased levels of endogenous CK and varied expression of genes involved in floral induction [46]. Rosebush (*Rosa Radrazz*) plants serve as a model in the research of light-mediated bud outgrowth [52]. Application of CK can induce bud outgrowth in both continuous darkness and light. Moreover, in the photocontrol process, CK is the first target of light. When CK is applied to cultivated buds in darkness, the amount of H_2_O_2_ in the buds falls to a level comparable to that seen in light [52]. In addition, this form of treatment initiates bud bursts and raises GSH levels. Treatment with the GSH synthesis inhibitor “buthionine sulfoximine” resolves the chain of events involving the metabolism of H_2_O_2_/GSH in the photocontrol process. This inhibited bud burst even in the presence of CK, indicating that the beneficial impact of CK on GSH is the first step in a chain of events which then triggers H_2_O_2_ scavenging and bud outgrowth [52]. A recent study revealed salicylic acid (SA) induces apple flowering by suppressing GA and stimulating IAA [53]. The researcher did not quantify CK content following SA treatment, providing avenues for future research. Additionally, research on the involvement of several TFs families such as WRKY, MYB, and AP2/ERF in floral induction is missing. These TFs families have been reported previously for regulating floral induction in several crops [54,55,56]. Based on the evidence, CK’s effect on the expression of floral induction TFs would be helpful in the identification of key genes for future breeding research.

#### 3.4.2. Flower Senescence

The senescence of a flower is commonly described when the petal wilts, weakens, or completely abscised [57]. A carefully controlled process, flower senescence demonstrates many of the molecular, biochemical, and structural alterations that characterize programmed cell death. Internal or external stimuli can initiate flower senescence, and phytohormones have been demonstrated to be essential in initiating and regulating the course of senescence, frequently through combinatorial interactions [57]. The senescence of many floral organs—including the petal, sepal, receptacle, stamen, and pistil—was accompanied by high levels of expression of *RhPR10.1* (*Pathogenesis related protein*), which was increased by exposure to ethylene [58]. Virus-induced gene silencing (VIGS) of *RhPR10.1* expression in rose plants accelerated floral senescence, resulting in increased expression of the senescence marker gene *RhSAG12* and a faster rate of ion leakage in the petals. *RhPR10.1*-silenced plants exhibited decreased CK content and the expression of three genes involved in the signaling pathway. Post-CK treatment, the *RhPR10.1*-silenced plants’ increased rate of petal senescence returned to normal [58]. In addition, it was shown that *RhHB6* (homeodomain-Leu zipper I) bound to the *RhPR10.1* promoter and that inhibiting its expression furthered floral senescence. The CK content increased by the ethylene-induced RhHB6-RhPR10.1 regulatory module may act as a brake on ethylene-induced senescence [58]. During the senescence of rose flowers, exogenous ethylene application significantly stimulated the expression of *RhERF3* [59]. *RhERF3* overexpression (OE) sped up the process of floral senescence, but transient silencing of the gene delayed it. In the *RhERF3*-OE line, the expression of the *IPT* gene was downregulated, but in silenced plants, the opposite pattern was observed. The expression of *9-cisepoxycarotenoid dioxygenase* (*RhNCED1*), the ABA biosynthesis gene, is induced when *RhERF3* directly binds to its promoter, suppressing the CK contents and causing senescence [59]. *RhNCED1-OE* petals exhibited partial or complete senescence after 14 days and displayed an elevated rate of ion leakage. The transcriptomic analysis is a fast and easy way to observe the expression of the trajectory of specific genes during flower senescence. The study of key senescence-related genes from the NAC transcription factor family is yet to be done. For example, *AtNAC029* was strongly upregulated during senescence, and its silencing considerably slowed the senescence process [60]. The response of these genes to CK application would further enhance our understanding of the CK-mediated flower senescence process.

#### 3.4.3. Sex Regulation

Since sex difference is a means of outbreeding and a primary source of genetic diversity, scientists typically find it a topic of great importance. Sex determination in flowering plants is a developmental process promoting allogamy to maintain survival and fitness [37,61,62]. Angiosperm sex determination systems have evolved independently many times, indicating various genetic and developmental processes involved in creating unisexual flowers [37]. Eight phases comprised the floral development of *Sacha inchi* (*Plukenetia volubilis*, Euphorbiaceae), with Stage 3 being the first morphological difference between the male and female flowers [63]. 6-BA successfully stimulated the growth of female flowers and the commencement of gynoecium primordia, particularly in the early phases of flower development [63]. The impact of CK on female primordia in *P. volubilis* flowers implies that CK has a feminizing role in determining sex [63]. Kiwifruit (*Actinidia chinensis*) is a woody perennial vine that is dioecious and has a long lifespan. To increase food security in the face of climate change, shorter generation times and the introduction of hermaphroditism might hasten crop development and enable alternative farming. The *Shy Girl* (*SyGl*) gene, which shares similarities with type-C cytokinin response regulators, may inhibit the formation of gynoecium in model plants [64]. Functional proof for *SyGl’s* participation in feminization suppression was provided by the quick blooming hermaphrodites with recovered gynoecia function and viable pollen that resulted from knocking out *SyGl* in *A. chinensis*’ *Bruce* (male kiwifruit) [64]. As a new player in sex determination, CK’s role can be further studied in some important species, such as cucumber, which is primarily monoecious. Applying CK to cucumber plants would be ideal for understanding its role in inducing femaleness.

### 3.5. Cytokinin Regulates Fruit Development

Due to its direct involvement in cytokinesis, CK is indispensable for fruit development. A series of studies have highlighted the direct participation of CK in fruit growth and fruit-related traits.

#### 3.5.1. Fruit Setting

**Figure 1:**
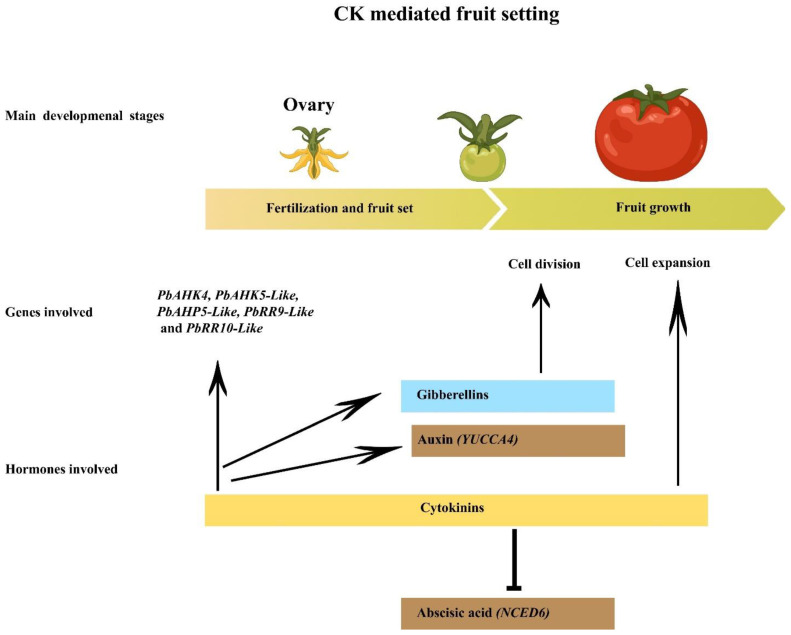
The graphic depicts the process of parthenocarpic fruit formation mediated by CK. Following the application of CK, genes involved in CK signaling are inducted, as are genes related to auxin and GA. Induced auxin and GA stimulate the process of cell division and enlargement by suppressing ABA (NCED6) in the unfertilized ovary, leading to the subsequent development of parthenocarpic fruit.

The involvement of CKs has been extensively highlighted in fruit formation across different horticultural crops [51]. A synthetic CK analog called N-(2-chloro-4-pyridyl)-N’-phenylurea (CPPU) can cause fruit set in a variety of horticultural crops [51]. In tomato, CPPU-treated ovary at the anthesis stage significantly increased the active CK contents and the mRNA level of CK biosynthesis genes [65]. In contrast, low CK levels and decreased CK biosynthetic gene expression were seen in tomato ovaries that were not fertilized and were treated with water, yet they were unable to bear fruit [65]. CKs accumulated more abundantly in cucumbers’ DDX (natural parthenocarpic line) than in the ZK (non-parthenocarpic line). The downregulation of the CK dehydrogenase genes *CKX1* and *CKX3*—together with the increased expression of the CK catabolic genes *CYP735A1*, *CYP735A2*, and *LOG1*—promotes vigorous parthenocarpic fruit set ratio in DDX [8]. In parthenocarpic tomato (RNAi-generated *TOPLESS1* (*SlTPL1*) gene lines, elevated levels of CK (DZ and iP) were observed [66]. In cucumber, the natural parthenocarpic line DDX and the CPPU-induced parthenocarpy in ZK showed increased transcription of the CK signaling gene *CsRR8/9b* [8,67]. In the ZK line, the (*CsRR8/9b*) genes drastically decreased in the absence of CPPU treatment; however, other CK signaling genes (*CsRR3/4a*, *CsRR8/9a*, and *CsRR8/9c*) were significantly increased [8,67]. This indicated that the *CsRR8/9a*, *CsRR8/9c*, *CsRR3/4a*, and *CsRR3/4b* genes had a detrimental impact on controlling cucumber fruit sets prior to fertilization. Comparably, in the *Ficus carica* L. plant, the unfertilized ovaries treated with CPPU that subsequently yielded parthenocarpic fig fruit showed persistently elevated expression of two CK signaling genes (*comp22053_c0* and *comp16589_c0)* [68]. In CPPU-treated flowers and receptacle tissue, the ABA biosynthesis *NCED* (*comp26438_c0*) and signaling *AFB* (*comp15261_c1* and *comp30505*) genes showed decreased expression [68]. Exogenous CPPU activated *PbAHK4*, *PbAHK5-like*, *PbAHP5-like*, *PbRR9-like*, and *PbRR10-like* genes in pears that modulate CK signaling [69]. It was discovered that the *PbRR9-like* gene binds to the promoters of *PbYUCCA4* and *PBNCED6*, stimulating *PbYUCCA4* and suppressing *PbNCED6* activity, respectively [69] (Figure 1). Augmented auxin production and reduced ABA under CK treatment may have contributed to the unfertilized ovary development that resulted in parthenocarpic pears [69]. Interestingly, the CPPU-mediated parthenocarpy in pear was independent of GA because the application of paclobutrazol (PAC), a GA inhibitor, did not compromise the parthenocarpic fruit setting [69]. More recently, the study of [70] revealed that GA does play a significant role in CPPU-induced parthenocarpy in melon. Post-PAC treatment, partial restriction of parthenocarpic melon fruit production was observed [70]. The above studies confirm that CPPU works through dissimilar pathways in different species during the fruit-setting stage. Additionally, the participation of CK signaling genes in parthenocarpic fruit formation is evident (Figure 1). However, the research is only limited to the exogenous application of CK to unfertilized ovaries that produce parthenocarpic fruit. Several researchers have performed RNA-sequencing of CK-treated unfertilized ovaries. Numerous TFs such as *SPL* and *NAC* associated with hormonal regulation, including CK, have been screened from the datasets [51]. Unfortunately, functional studies of how these TFs modulate the CK-mediated fruit setting have yet to be performed. These TFs could be taken as biological macromolecules and reduce the farmer’s dependency on exogenous CK application.

#### 3.5.2. Fruit Size and Length

Fruit size and length are key components that determine the fruit’s marketability. Several studies have reported CK modulating fruit size and length. The greening of spathe post-anthesis in the ornamentally important herbaceous plant peace lily (*Spathiphyllumwallisii Regel*) resulted in enlarged fruit [71]. The biochemical analysis of fertilized green spathe revealed a higher accumulation of t-ZR [71]. *Actinidia chinensis* (Chinese kiwi), when treated with CPPU, causes an increment in the weight and length of the fruit [72,73]. The expression of *PIP2.4*, an aquaporin gene, was induced significantly in the CPPU-treated fruitlets (28 DAA), which drove the uptake and accumulation of water and glucose and spawned larger kiwifruit [72]. The transgenic tomato plants overexpressing the *AtCKX2* gene (CK-inactivating enzyme) produced smaller fruit than WT [74]. The suppression in endogenous CK, auxin, and GA accompanied by hampered cell division was observed in the *AtCKX2*-overexpressed tomato lines. The downregulated expression of *Cyclin U1;1 (CYCU1;1)*, *Expansin 2*, and *GA20ox1* (GA biosynthesis) and upregulated expression of *GA2ox5* (GA inactivation) and *LAX3* (auxin influx carrier) was also noted in *AtCKX2*-overexpressed lines [74]. The silencing of the *cryptochromes (SlCRYs)* gene in tomato resulted in enlarged fruit by inducing endogenous CK [75]. On the contrary, overexpression of the *CRY* gene increased the expression of cytokinin oxidase genes *CKX1*, *CKX2*, *CKX3*, *CKX4*, *CKX5*, and *CKX6*, which further inhibited the accumulation of CK and led to smaller fruit. The overexpression of the *VvPUP1* gene in *Vitis vinifera* positively regulates CK transport [76]. The improved CK transport further intensified sugar accumulation and cell division, promoting berry enlargement [76]. The small-auxin-up-regulated RNA (SAUR) gene family consists of around 60–140 members in most higher plant species. These genes are highly responsive to auxin and other hormones, including CKs [77]. The *BhSAUR60* is highly expressed in wax gourd (*Benincasa hispida*) fruit, and overexpression led to longer fruit [78]. The ovaries of *BhSAUR60*-overexpressed plants have low expression of the *CKX7* gene, which could be the reason for elongated fruit [78]. Cell expansion is a complex process generally governed by an array of transcription factors such as *Expansins* [79,80]. Despite the presence of expression study of several expansins genes under CK treatment during fruit enlargement, molecular approaches should be instilled to understand the converging role of these expansion-related biomolecules with CK genes.

#### 3.5.3. Fruit Attributes

Pre and postharvest fruit traits are of key significance importance that define the fate of fruit marketability [81,82]. Enlarged and heavyweight fruit put pressure on the fruit pedicel, thus making the pedicel a critical trait. The study of [83] identified a key gene controlling the pedicel length in melon (*Cucumis melo* L.). The long pedicel (M125) and short pedicel (X055) melon lines were put through a forward genetic screen, and via bulk segregant analysis (BSA) and quantitative trait loci (QTL) mapping, a gene responsible for pedicel length was narrowed down to an 89 Kb region on chromosome 3. *MELO3C010972*, encoding cytokinin oxidase, was mapped as the candidate gene with high expression in X055 compared to M125 [83]. It can be assumed that *MELO3C010972* restraining CK accumulation in the pedicel thus confers short pedicel phenotype in the X055 melon line. Warty cucumber fruit is a significant quality characteristic that substantially impacts the fruit’s look and market value. The study of [84] discovered that cucumber fruit skins, comprising the spines and tubercules, have high expression levels of the basic Helix-Loop-Helix (bHLH) gene *HECATE2* (*CsHEC2*). While overexpression of *CsHEC2* resulted in increased wart density and CK level, *CsHEC2* knockout using the CRISPR/Cas9 technology reduced wart density and CK accumulation in the fruit peel. The CK *hydroxylase-like1* gene (*CsCHL1*) catalyzes CK biosynthesis and is directly bound by *CsHEC2*. *GLABROUS3* (*CsGL3*, a crucial spine regulator) and Tuberculate fruit (*CsTu*, a core tubercule development factor) were physical partners of *CsHEC2*, and these interactions improved *CsHEC2*-mediated *CsCHL1* expression even more [84]. A previous study by [85] stated that instead of CK, the level of endogenous auxin significantly changed in the cucumber *CsTS1* gene overexpression lines, which displayed a phenotype similar to that of *CsHEC2* overexpressed lines. It can be suggested that CK works independently of auxin during the tuberculate formation; however, further explanation is required. Fruit cracking is a kind of morphological disorder that seriously affects marketability. Applying CK (20 mg L^−1^) to the “Pink Lady” apple cultivar in the early stage of fruit development strengthens its peel by triggering the transcription of cuticle biosynthesis and cell wall-related genes [86]. Induced transcriptional activity of cuticle biosynthesis genes (*ALIPHATIC SUBERIN FERULOYL-TRANSFERASE*, *ECERIFERUM3*) strengthens the peel and denies the cracking incidence [86]. In tomato, compared to the Japanese cultivar, the boosted expression of *Sl-IPT3* and *Sl-RR16/17* encouraged the local biosynthesis of iP in hypocotyl, further stimulating the transport of minerals and nutrients to shoots in the Dutch cultivar [87]. The pronounced assimilated transport assisted the xylem development and resulted in higher fruit yield of Dutch cultivar [87]. Fruit curving is a major problem in horticultural crop production that significantly damages the marketability of the crop. In cucumber, high ethylene contents cause fruit curvature and, in some cases, fruit drop [88]. CK application to cucumber ovary suppresses ethylene production and could be a useful strategy for producing straight and normal-sized fruit.

**Table 1:** Studies enlisting the role of cytokinin regulating organ development in horticultural crops.

Organs	Crops	Gene	Functions	References
Roots	*Malus domestica*	*MdRR12* ↑	Negative regulator of AR formation	[34]
Leaf	*Solanum lycopersicum*	*SlIPT2* and *SlIPT4 ↓*	silencing of *SlIPT4* resulted in premature leaf senescence	[40]
Shoot	*Solanum tuberosum*	*vacuolar invertase ↑*	Regulation of bud outgrowth	[43]
	*Cucumis sativus*	*CsCKX ↑*	Extremely short internodes	[44]
	*Solanum lycopersicum*	*SlRR6 ↑*	Plant height regulation	[45]
	*Malus domestica*	*MdIPT1 ↑*	*MdIPT1*-*OE* showed a multi-branching phenotype	[46]
Flower	*Dimocarpus longan*	*FLOWERING LOCUS 1 ↑*	Increased CK concentration in the shoot induces flowering.	[48]
	*Malus domestica*	*AHK*, *AHP*, and *ARRs ↑*	Floral induction	[49]
	*Rosa hybrida*	*RhPR10.1 ↓*	*RhPR10.1* accelerated flower senescence	[58]
	*Rosa hybrida*	*RhERF3 ↑*	Overexpression of *RhERF3* impeded CK accumulation and hastened senescence	[59]
	*Actinidia chinensis*	*SyGl ↓*	block gynoecium development	[64]
Fruit	*Cucumis sativus*	*CYP735A1*, *CYP735A2*, and *LOG1 ↑*	Promote fruit development	[8]
	*Pyrus communis*	*PbRR9-like*, and *PbRR10-like ↑*	Parthenocarpic fruit induction	[69]
	*Solanum lycopersicum*	*AtCKX2 ↑*	Overexpression produced smaller tomato fruit	[74]
	*Vitis vinifera*	*VvPUP1 ↑*	Improved CK transport further intensified cell division, promoting berry enlargement	[76]
	*Cucumis melo*	*MELO3C010972 ↑*	Short pedicel phenotype	[83]
	*Cucumis sativus*	*CsCHL1 ↑*	Warty fruit phenotype	[84]

*↑* = represent upregulation, ↓ represent downregulation.

## 4. Cytokinin in Stress Biology

Under extended periods of abiotic and biotic stresses, alteration of endogenous CK levels is widely observed in plant tissues [89]. Here, we focus on the role of CK in horticultural plants under different abiotic and biotic stresses.

### 4.1. Abiotic Stresses

#### 4.1.1. Cold Stress

Cold stress (CS) is a significant abiotic stress that causes chilling, further arrests growth, and restricts yield [90]. Phytohormones such as CK have been reported to alleviate the negative effect of cold stress. When overexpressed in apple calli and tomato plants, *MdIPT5b*—a CK biosynthesis gene—magnifies the cold tolerance [91]. The *IPT5b*-overexpressed plant accumulated proline in abundance while fortifying the redox and osmotic balance during low temperatures, which could be the reason for vitalizing cold tolerance [91]. Despite the compelling proof, few studies presented CK in cold stress at the physiological stage. Most of the horticultural crops (vegetable crops in particular) are vulnerable to low temperatures; therefore, research investment is required to address this essential abiotic stress in detail. Examining the cold stress marker genes in response to CK treatment or applying deep molecular approaches to understand the complex pathway would be beneficial, as well as fine-tuning the horticultural plant immunity to cold stress. For instance, the HSP transcription factors have been reported to increase tolerance against cold stress [92]. AP2/ERF TFs act upstream of the COR/RD (cold-regulated/responsive to dehydration) genes [93]. These key TFs could be of great interest in investigating their interactive role with CK signaling genes. Another aspect of CK synergistic or antagonistic relation can be studied following cold stress. Since hormones work in a complex wired-up network, studying their crosstalk in horticultural plants under cold stress would greatly enhance our understanding.

#### 4.1.2. Heat Stress

**Figure 2:**
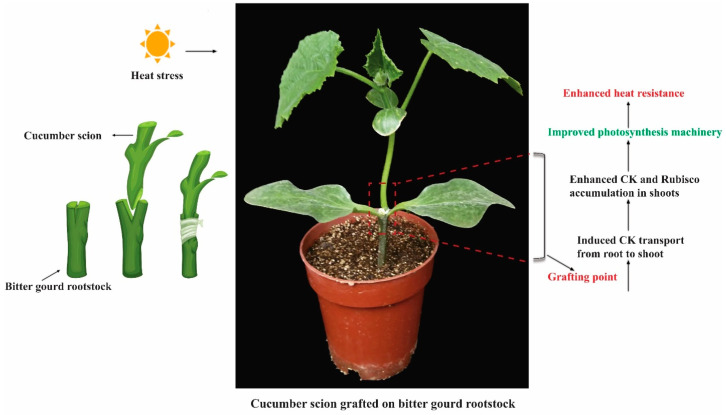
An illustration presenting the role of CK in heat stress in cucumber. The rootstock of bitter gourd and scion of cucumber was used to understand. The uptake of CK from roots to shoots enhances the CK and rubisco accumulation. The enhanced CK accumulation improved photosynthesis machinery, thus improving heat tolerance.

Surface and air temperatures are rising due to global climate change events. Plants experience heat stress when temperatures exceed the ideal range for healthy development [94]. This damages plant development negatively and irreversibly, endangering global food security by drastically lowering crop overall output [37]. It is reasonable that CK will increase the heat stress tolerance of horticultural plants. For example, the roots of grafted cucumber plants on bitter gourd (*Momordica charantia*) rootstocks were exposed to heat [10]. Cucumber scions’ resistance to heat stress was increased by bitter gourd rootstock. This improvement was positively linked with greater levels of CK in the root and leaf sections. Furthermore, enhanced CK transport from roots in grafted plants under high temperatures was suggested as the reason for the greater CK and Rubisco levels in the shoots of cucumber scion compared to the shoots of self-grafted cucumber plants. According to these findings, CK moved from the bitter gourd rootstock to the scion and caused Rubisco to accumulate in the leaf, enhancing the plants’ ability to withstand heat [10]. No study thus far is available to increase our knowledge regarding CK involvement in heat stress biology. Some examples from *Arabidopsis* highlighted the positive impact of CK in diminishing the negative impact of heat stress. The application of INCYDE-F (inhibitor of cytokinin oxidase/dehydrogenase) significantly increased the *trans*-zeatin contents and thus promoted tolerance to heat stress [95]. A model presented the role of CK in regulating cucumber response to heat stress (Figure 2).

#### 4.1.3. Hypoxia/Waterlogging

Floods are the worst natural calamity that jeopardizes agricultural and food security globally after droughts. Between 2008 and 2018, losses from floods were USD 21 billion, or 19% of all agricultural losses in low- and lower-middle-income nations [96]. Limited gas diffusion results in oxygen (O_2_) deprivation in flooded plants, with oxygen availability falling from 21% (normoxia) to less than 1% when submerged (hypoxia) [96]. Plants utilize intracellular signals such as dynamic fluctuations in O_2_, ROS (reactive oxygen species), NO (nitric oxide), and ethylene to adapt to flooding during submergence [97]. In contrast to non-submerged tissues, ethylene can be trapped in plants by inhibited gas diffusion in water, which can result in concentration rises of up to 20 times within the first hour of submergence [98,99]. Pretreatments with ethylene improved the model plant *Arabidopsis’s* ability to withstand hypoxia, rosette sizes, and root tip survival by enabling the tip to regrow after recovery [100]. Nevertheless, it is still unknown what mechanisms underlie CK-mediated hypoxia survival. As was previously indicated in the section on root biology, CK often limits the production of AR in horticultural crops. When a plant is stressed by waterlogging, AR formation is crucial [101]. Thus far, no study has been available to give a clear verdict on the CK position in plant response to waterlogging stress. Recently, several investigations have indicated a reduction in leaf tZ and DHZ concentrations after submergence and waterlogging in soybean and tomato, respectively [102,103]. The decrease in CKs could mitigate the suppression of ethylene- and auxin-induced cell elongation, resulting in epinastic bending [102]. The *CKX2* exhibits significant upregulation in the ABA-deficient mutant, indicating CK degradation in waterlogged tomato roots [104]. Notable reductions in the expressions of *CYP735A1* and *CYP735A2* at 24 and 48 h of root waterlogging, respectively, signify less tZ synthesis in roots [105]. Transcriptome analysis of grapevine roots subjected to 12 h, 24 h, and 96 h of hypoxia demonstrated overexpressions of *IPT* and *RRAs*, whereas positive regulators, *RRBs* and *HPPs*, were generally downregulated [105]. In summary, we can ascertain that CK levels diminish in the shoot under flooding stress, which may be ascribed to either a decrease in local CK synthesis in the shoot or a diminished CK influx into the leaves resulting from lower CK production in the roots. Owing to that, it would be interesting to see the crosstalk of CK with other hormones (ethylene and auxin) that have already been reported for waterlogging tolerance in plants.

#### 4.1.4. Salt Stress

Salt stress, or salinity, is the second most significant abiotic factor impacting global agricultural output due to its detrimental effects on a variety of physiological, biochemical, and molecular processes [106,107,108,109,110]. The antioxidant defense system, ion homeostasis, and the production of many phytohormones and osmoprotectants are all impacted by salinity [37,108,111,112]. Despite its indispensable role in growth activities, CK displays a mixed response to salinity. For example, the *CKX1* gene degrades active CK and increases the accumulation of secondary metabolites in response to salt stress [113]. Similarly, in *Physcomitrella patens*, overexpression of *PpCKX1* lessened the CK levels and increased salt tolerance [114]. Conversely, the salt-tolerant genotype of pepper exhibited reduced levels of CKX and increased levels of CK when cultivated in saline conditions [115]. The *SlIPT3* overexpressed tomato plants showed tolerance to salinity via elevated active CK contents, upregulation of CK signaling genes (*SlARR1*, *SlARR4*), sodium (Na^+^) and potassium (K^+^) ion homeostasis and revitalized photosynthetic activity [116]. The growth of cucumber plants was significantly cumbered when subjected to 50 mM NaCl stress [117]. The NaCl treatment raised the level of root CK at the beginning of stress. Adding silicon (Si) 0.3 mM to the saline growth medium alleviated the adverse effects of NaCl stress by inhibiting the accumulation of CK. The Si treatment lowered the level of root CK at 3, 6, and 9 days; however, it induced it after 12 days of NaCl stress. Higher proline activity and expression of CKX genes (*Csa4G647490* and *Csa1G589070*) were also observed in Si-treated plants [117]. Salinity stress generally causes leaf senescence by impeding the plant’s photosynthetic process. The Si treatment palliated the salinity-induced leaf senescence by inducing the transcription of CKs (*IPT3* and *IPT6*) genes, enhancing the chlorophyll accumulation, suppressing the malondialdehyde (MDA) and senescence marker genes (*SAG15* and *WRKY53*) in tomato plants. In contrast, the *ipt1*,*3*,*5*,*7* mutant failed to limit the salinity-stimulated leaf senescence [118]. Higher CK contents were detected in the *Alq* tomato mutant, which produced parthenocarpic fruit when grown in saline conditions [119]. The apple rootstock “robusta” exhibited a sustained elevation in CK levels when subjected to salt stress [120]. By examining the genes responsible for CK production and breakdown, the researcher discovered that the significant upregulation of *IPT5b* in robusta roots had a role in sustaining the elevated amounts of CK. A 42 bp deletion in the *IPT5b* promoter region increased its expression levels and promoted salt tolerance in robusta × M.9 segregating populations. The 42 bp deletion deleted a proline response element (ProRE), which reportedly negatively affects *IPT5b* expression in response to proline. The robusta cultivar improves salt tolerance by maintaining high CK levels under salt stress. This is because the deletion of the ProRE prevents proline from inhibiting the production of *IPT5b* [120]. A very recent study in maize elaborated on the role of CK in maintaining plant health under salinity stress [121]. The researcher stated that the excessive buildup of chloride (Cl^−^) in the aboveground tissues when exposed to saline environments has a detrimental effect on crops. Enhancing the exclusion of Cl^−^ from shoots enhances salt tolerance in different crops. The type-A response regulator (*ZmRR1*) controls Cl^−^ exclusion from shoots and contributes to natural salt tolerance in maize. *ZmRR1* may prevent His phosphotransfer (HP) proteins from mediating CK signaling and salt tolerance. A naturally occurring non-synonymous SNP mutation promotes *ZmRR1-ZmHP2* interaction, giving maize plants a salt-hypersensitive phenotype [121]. In the wild type, *ZmRR1* experiences degradation in the presence of salt, resulting in the release of *ZmHP2* from the inhibitory effects of *ZmRR1*. Consequently, *ZmHP2*-mediated signaling enhances salt tolerance principally by facilitating the exclusion of Cl^−^ from shoots [121]. Based on the evidence provided above, CK’s role in regulating plant tolerance to salinity is still conflicting, thus providing avenues for future research.

#### 4.1.5. Drought Stress

Drought stress (DS) is a dangerous natural hazard to crop production, influencing a considerable fraction of the world population, primarily those residing in arid and semi-arid areas [122,123,124]. Therefore, to ensure food security, addressing DS is the need of the day. The *MdDREB6.2* overexpression prompts the mRNA level of *MdCKX4a*, thus enhancing DS tolerance [125]. The increased root hydraulic conductivity, stomata closure, and decreased CK accumulation were the causes of the increased DS tolerance [125]. The application of 20 µM melatonin over creeping bentgrass (*Agrostis stolonifera*) significantly reduced the adverse effects of drought stress by upregulating the expression of CK biosynthesis (*IPT*) and signaling genes (*AsRR4*, *AsRR9*, *AsRR10*) [11]. The melatonin-treated plants also delayed the drought-induced leaf senescence by enhancing the transcription of *JUB1* and *DREB2a* (dehydration-responsive genes) while arresting the *Chlase*, *PPH*, and *Chl-PRX* (chlorophyll-degradation genes) [11]. Dehydration-induced senescence is highly important to maintaining the aesthetic characteristics of *Rosa hybrida.* The work of [126] revealed that CK degradation plays a vital role in the dehydration tolerance of *Rosa hybrida* via petal senescence. The genes *RhNAP* and *RhCKX6*, triggered by DS, also stimulated the senescence of mature petals. Molecular analysis showed that *RhNAP* binds to and induces the expression of *RhCKX6*, leading to CK degradation. The silencing of either *RhNAP* or *RhCKX6* crippled the tolerance of *Rosa hybrida* to DS. The reduced tolerance level due to high CK accumulation in *RhNAP* or *RhCKX6* antisense plants delayed leaf senescence. Delayed senescence of mature petals impeded the growth of young petals, thus causing dehydration sensitivity [126]. Therefore, CK degradation via the *RhNAP-RhCKX6* module is essential for young petals to thwart DS. Based on the studies above, CK’s role in DS is puzzling and can easily be misunderstood, particularly by early-stage researchers. To understand the perplexing part of CK in regulating the plant response to DS was explained by [127]. The overexpression of the *MdIPT5b* gene aggravates the tolerance of apple callus and tomato seedlings to DS via CK homeostasis. The transcriptional repressor complexes *MdbZIP2*, *MdbZIP39*, and *MdbZIP80* bind to the promoter region of *MdIPT5b* and suppress its expression, increasing the susceptibility of apple callus and tomato seedlings to DS. In contrast, the RNAi silencing of *MdBZIP80* triggered the expression of *MdIPT5b* and progressed drought tolerance in apple callus and tomato seedlings [127]. To further confirm whether or not *MdIPT5b* is responsible for drought tolerance in *MdBZIP80* RNAi plants, the co-suppression silencing assay was performed. The co-suppression of *MdBZIP80-MdIPT5b* restored the drought sensitivity of apple callus and tomato seedlings [127]. The *MdIPT5b* suppresses *MdbZIP2*, *MdbZIP39*, and *MdbZIP80* modules to maintain growth under DS conditions. While the study clearly suggests CK’s positive role in DS tolerance, it is still unclear because the role changes from species to species. There may be a conserved pathway regulating the growth and DS at the same time and therefore need further exploration. Additionally, ABA is a key hormone in regulating plant response to drought. The antagonistic crosstalk between CK and ABA has been investigated in Arabidopsis [128]. The *sucrose nonfermenting1-related kinases SnRK2.2*, *SnRK2.3*, and *SnRK2.6*, which are pivotal kinases in the ABA signaling pathway, directly interact with and phosphorylate type-*A response regulator 5 (ARR5*), a negative regulator of CK signaling. The phosphorylation of Ser residues in *ARR5* by *SnRK2s* increased the stability of the *ARR5* protein. Plants that overexpress *ARR5* exhibited hypersensitivity to ABA and enhanced drought tolerance, which could not be replicated by overexpressing a non-phosphorylated *ARR5* mimic. Furthermore, the type-B *ARRs—ARR1*, *ARR11*, and *ARR12*—physically interact with *SnRK2s* and inhibited the kinase activity of *SnRK2.6*. The *arr1*,*11*,*12* triple mutant demonstrated heightened sensitivity to ABA. Genetic research revealed that *SnRK2s* function upstream of *ARR5* and downstream of *ARR1*, *ARR11*, and *ARR12* in the regulation of ABA response and drought tolerance [128]. The CK–ABA crosstalk could be studied in horticultural plants, which will further enhance our understanding of this complex and important stress.

#### 4.1.6. Heavy Metals

Heavy metals are increasingly becoming troublesome for horticultural plant growers. Heavy metals damage plant health and intoxicate the soil and groundwater [129,130,131]. The review of [129] thoroughly explained the role of phytohormones in regulating the response to heavy metal stress; however, the author missed out on representing the crucial role of CK. The 10 μM exogenously spraying CK to eggplant (*Solanum melongena* L.) limited cadmium toxicity and boosted photosynthesis and antioxidant machinery [132]. In soybean, the *IPT* transcript level was up-regulated after exposure to Cd, leading to increased CK accumulation and augmented heavy metal tolerance [133]. Kinetin is a CK with phytoextraction-enhancing capabilities in sites contaminated with heavy metals. The significant up-regulation of the kinetin 9-riboside content in grape leaves under Low-ECS (low excess copper stress) was caused by the increased expression of CK synthesis genes/proteins and inhibition of CKs degrading genes at the transcription and translation level [134]. These results suggest that a low-ECS can enhance the buildup of CK, hence facilitating cell proliferation and the movement of copper in grapevine leaves, which in turn enhances tolerance [134]. The CK application to horticultural crops subjected to heavy metal stress (cadmium and magnesium) should be studied in detail. There is a need for extensive research that applies molecular and physiological approaches. CK biosynthesis and signaling genes could be considered for research, particularly in leafy vegetables, which are more susceptible to toxic heavy metals. Additionally, the tango between CK and others can be studied. For instance, methyl jasmonate (MeJA) plays a crucial role in improving plant tolerance to heavy metals by reducing their accumulation and by coordinating the ion transport system, antioxidant enzyme activities, and chelating capacity in plants [135]. The study of [136] advocated the synergistic role of CK and MeJA. The synergy between CK and auxin has been reported numerous times [137,138]. Auxin participation in regulating plant tolerance to heavy metals has been explained in several crop species [139]. All the aforementioned references pave the way for a worthy investigation of how CK-other phytohormones crosstalk regulate the response of horticultural crops to heavy metals.

### 4.2. Biotic Stresses

#### 4.2.1. Nematodes

**Figure 3:**
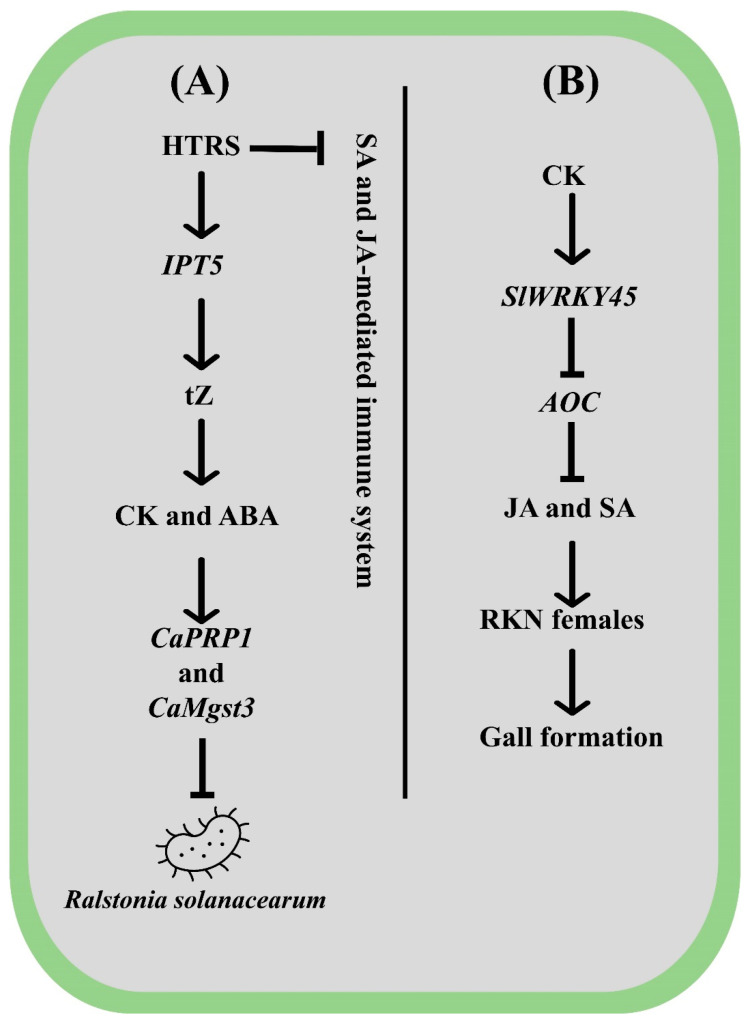
The graphical illustration presents CK’s mechanistic role in mediating plant response to Meloidogyne and Ralstonia solanacearum stress. (**A**) Under high-temperature Ralstonia solanacearum (HTRS), the plant shutdown the SA and JA-mediated immune response while activating the CK. The IPT5 gene stimulates the CK and ABA biosynthesis and expression of CaPRP1 and CaMgst3, conferring tolerance to Ralstonia solanacearum. (**B**) The application of CK induces the transcription of SlWRKY45, which suppresses the accumulation of JA and SA and—in the process—causes susceptibility to RKN (Meloidogyne).

As members of the sedentary endoparasitic nematode group, plant-parasitic nematodes need to develop and maintain NFS (nematode feeding sites), also known as giant cells (root-knot nematodes, RKN; *Meloidogyne*) or syncytium (cyst nematodes, CN; *Heterodera* and *Globodera* spp.) [140]. These galls impair the roots’ regular physiological processes, obstruct the flow of nutrients and water, slow down host development and yield, and sometimes even cause the host to expire [37,141]. Because CK signaling is essential for controlling RKN disease, there is conjecture that CKs produced by worms may be injected into plants to aid parasitism [142]. These findings were further supported by [143], who provided genetic evidence that nematode-derived CK activates the host cell cycle during infection. The study of [144] found that *Arabidopsis* lines with reduced CK sensitivity showed minimal susceptibility to nematode infection, indicating that CK signaling is required for optimal nematode development. In tomato roots, the transcription level of *SlWRKY45* was increased in response to CK and SA but not to jasmonic acid (JA) [145]. The roots of overexpressed *SlWRKY45* tomato plants had a higher number of RKN females than WT. The expression of cytokinin response factors (*CRF1*, *CRF6)*, JA and SA marker genes, proteinase inhibitor (PI), and *pathogenesis-related protein (PR1)* was significantly impeded in the *SlWRKY45* overexpressed plants. This suggests that *SlWRKY45* promotes tomato susceptibility by strengthening the CK and RKN synergy [145]. A more detailed explanation of *SlWRKY45*-mediated tomato immune response to RKN was presented by [146]. The *SlWRKY45* binds to the promoter of *ALLENE OXIDE CYCLASE (AOC)* (JA biosynthesis gene) and, in the process, represses its expression. The reduced accumulation of JA attenuates tomato resistance to RKN [146]. The *slwrky45* mutants, on the other hand, showed fewer gall and egg counts per gram of roots than the natural type. Although the author did not quantify the CK activity, based on the report of [145], it can be assumed that *SlWRKY45* triggers CK accumulation while restraining JA and SA, encouraging gall formation (Figure 3B). However, there are still unanswered questions that need to be addressed in the field of plant-nematode biology. Following CK treatment, a high throughput transcriptomic analysis should be utilized to understand the complex molecular pathway underlying horticultural crops’ response to the nematode.

#### 4.2.2. Fungal Stress

**Figure 4:**
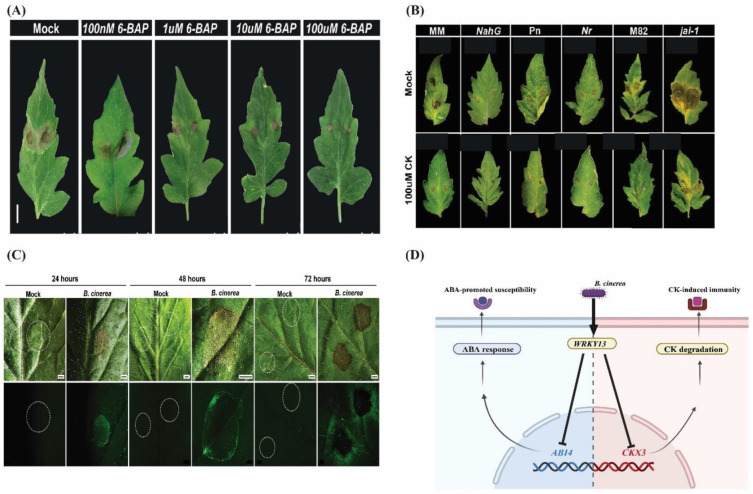
Graphics presenting the CK-mediated immune response against fungal stress in rose. (**A**) Increasing the dose of CK (6-BAP) diminishes the pathogenic effect of *B. cinerea*. (**B**) Leaves of different hormonal mutants treated with *B. cinerea* and 6-BAP (**C**) pTCS::3 ×VENUS (stereomicroscope) analysis of expression surrounding *B. cinerea* inoculation site at indicated time points after droplet inoculation. Plants treated with 1 μM NaOH were used as mocks. The pictures were generously provided by Professor Maya Bar [13]. (**D**) CK treatment to rose petals increases the resistance by inducing the transcription of the RhWRKY13 gene. The RhWRKY13 further suppresses the CKX3 and ABI4, thus reducing CK degradation and inducing resistance.

Many fungal diseases damage horticulture crop productivity [147]. *Botrytis cinerea* (*B.cinerea*) is a devastating grey fungal mold affecting more than 200 plant species [147]. Plant response to *B. cinerea* is often associated with SA, JA, and ethylene (ET) (usually known as immune-responsive hormones). However, developing stories about CK inducing resistance against *B.cinerea* have been reported recently. For example, the study of [13] observed resistance in tomato against *B.cinerea* in CK-treated plants. The exogenous application of CK activated the PRR (cellular trafficking of the pattern recognition receptor) LeEIX2, aid tolerance to *B. cinerea* in an SA- and ET-dependent manner but independent of JA. CK induces the expression of Pto-interacting 5 (*Pti-5*), pathogenesis-related proteins (*PR1a*, *PR-1b*), and pathogen-induced 1 (*PI-1*). Interestingly, the two-component signaling sensor (TCS) fused the VENUS fluorescent protein as a reporter, thus indicating that the CK signaling pathway is activated upon *B. cinerea* infection in mature leaf tissue. Quantifying TCS-driven VENUS fluorescence in transgenic M82 tomato plants showed that the TCS-driven VENUS signal is significantly higher after 24–96 h of *B. cinerea* inoculation than the mock-treated plants. *B. cinerea* inoculation causes a specific response in the leaf tissue, following the spread of infection and necrosis of the tissues. Additionally, the CK-responsive VENUS halo spreads out from the site of pathogen inoculation. Mock-treated leaves produced little to no TCS-driven VENUS signal [13] (Figure 4). It can be suggested that CK is crucial for activating plant defense machinery upon pathogen infection. More recently, the expanded understanding of CK-mediated resistance to *B. cinerea* in *Rosa hybrida* has been provided by [148]. The application of CK progressed the resistance in rose petals against *B. cinerea.* The transcriptional repressor gene *RhWRKY13* displayed upregulated expression post-CK treatment. *RhWRKY13* binds to and inhibits the expression of *RhCKX3* and *RhABI4 (ABA INSENSITIVE4)*. Silencing of *RhWRKY13* through virus-induced gene silencing (VIGS) heightened the susceptibility of rose petals to *B. cinerea.* Asymmetrically, restoration of a protective role of *RhWRKY13* was observed following CK treatment to *TRV*-*RhWRKY13* rose petals [148]. Thus, it can be concluded that CK concurrence with *RhWRKY13* positively regulates the immune response of *Rosa hybrida* to *B. cinerea* by suppressing the ABA and CK degradation. The mechanistic representation of CK-mediated resistance to *B. cinerea* is given in (Figure 4). Additionally, applying CK to tomato plants augmented resistance against *Oidium neolycopersici* (highly polyphagous powdery mildew fungus) similar to *B.cinerea* [13]. The boost in defense against anthracnose disease caused by *Colletotrichum gloeosporioides* in apple was observed following CK treatment [149]. The study used “Hanfu” diploid and autotetraploid apple as plant material for *Colletotrichum gloeosporioides* treatment. The hormonal quantification assay showed that the autotetraploid apple has higher endogenous CK content and better resistance against *Colletotrichum gloeosporioides* than diploid. The overexpression of *MdIPT8* significantly improved the resistance of apple against the *Colletotrichum gloeosporioides* [149]. On the other hand, overexpression of *AtCKX2* impairs the susceptibility of Micro-Tom to *Moniliophthora perniciosa* (a fungal pathogen responsible for causing witch’s broom disease) [150]. Overall, it can be suggested that CK can provide protection against an array of fungal diseases without restricting plant growth and could be used as a potential fungicide in the horticultural crop industry.

#### 4.2.3. Bacterial Stress

Bacteria pathogens are notorious for transmitting highly destructive diseases in horticulture plants. Among them, Citrus canker caused by *Xanthomonas citri* subsp. *Citri* (*Xcc*) is a severe bacterial disease affecting most commercially important citrus cultivars. The association of CK with citrus canker disease has been studied by [151]. The gene *CsLOB1* (*Lateral Organ Boundary 1*) was triggered following the infection with *Xcc.* The canker-sensitive citrus line “wanjincheng” was used as an explant for a transgenic experiment. The citrus plants overexpressing *CsLOB1* showed increased susceptibility to *Xcc.* The overexpression of *CsLOB1* in plants has resulted in the upregulation of *UGT85A2*, a *UDP-glucosyl transferase* that controls the N-glucosylation of CK. The high expression of *UGT85A2* further stimulated the transcription of CK-dehydrogenase genes and, in the process, suppressed CK accumulation. Contrariwise, *CsLOB1* antisense plants displayed enhanced resistance against *Xcc* [151]. Therefore, it can be assumed that average CK accumulation is vital for protection against the *Xcc.* Bacterial wilt is a destructive soil-borne disease affecting pepper plants caused by the *Ralstonia solanacearum* [152]. The interaction of *Ralstonia solanacearum* with plants is often affected by environmental changes either positively or negatively. The study of [153] addressed this issue by evaluating the pepper plant’s immune response to *Ralstonia solanacearum* under high temperature and high humidity (HTHH). Upon *Ralstonia solanacearum* infection under HTHH, the SA and JA-mediated immune system was shut down. Owing to that, the plant shifted to a CK-mediated immune response as the endogenous trans-Zeatin content and higher expression of *CaIPT5* were recorded under HTHH. Silencing of *CaIPT5* substantially compromised the resistance of pepper plants to *Ralstonia solanacearum* under HTHH. To further confirm the notion, the exogenous application of trans-Zeatin to tomato and tobacco intensified their resistance to *Ralstonia solanacearum* by amplifying the endogenous CK and ABA contents and triggering the expression of *CaPRP1* and *CaMgst3* [153]. The gene *CaPRP1* encodes a putative cell wall proline-rich glycoprotein [154], whereas the *CaMgst3* belongs to the plant glutathione S-transferases family are key in regulating pepper response to the pathogen [155]. The mechanism of CK-mediated defense response to *Ralstonia solanacearum* under high temperature and humidity has been presented in (Figure 3A). High humidity and fluctuating temperature are significant issues in protected horticulture as they propel the growth of several bacterial pathogens that further cause wilt disease. CK could be considered a potent bactericide in protected horticulture, particularly in the case of *Solanaceae* crop cultivation.

#### 4.2.4. Insect Resistance

CK’s role in regulating plant response to insect infestation has been addressed in several species except horticultural plants. For instance, the brown planthopper (BPH) and white-backed planthopper (WBPH) are the most damaging insect pests, significantly threatening rice output in Asia [156]. Map-based cloning allows functional investigation of *Bph6*, a gene that imparts resistance to planthoppers in rice [157]. *Bph6* encodes an uncharacterized protein that localizes to exocysts and interacts with the exocyst subunit *OsEXO70E1*. The expression of *Bph6* enhances exocytosis and contributes to the preservation and fortification of the cell wall. A synchronized signaling pathway involving CK, SA, and JA is activated in *Bph6*-bearing plants, which exhibit extensive resistance to all evaluated BPH biotypes and to WBPH without compromising yield, as these plants demonstrated sustained high performance in a field severely infested with BPH [157]. In another study, the external application of CK substantially enhanced rice tolerance to BPH. Augmenting endogenous CK through the knockout of cytokinin oxidase/dehydrogenase (*OsCKXs*) resulted in improved resistance to BPH [158]. Furthermore, the concentrations of the plant hormone jasmonic acid (JA) and the expression of JA-responsive genes were increased by CK treatment and in *OsCKX*--knockout plants. Moreover, the JA-deficient mutant *og1* exhibited increased susceptibility to BPH, and CK-induced BPH resistance was diminished in *og1*. The results demonstrate that CK-mediated BPH resistance is contingent upon JA [158]. Despite the evidence, the potent role of CK in increasing horticultural plants’ resistance against herbivores is missing.

#### 4.2.5. Genome Editing of CK Pathway Genes

The increasing number of articles presenting the efficacy of genome editing technologies such as CRISPR/Cas9 [159]. Thus far, the application of CRISPR/Cas9 in editing the CK pathway in horticultural crops is lacking. There are significant pieces of evidence for using CRISPR technology to enhance crop production and tolerance. For instance, the knockout of *OsCKX* not only improved resistance to BPH but also rice growth [158]. We believe modulating the expression of the CK pathway by editing them directly or their upstream TF could be useful in achieving bumper crop yield.

## 5. Conclusions and Perspectives

Cytokinin (CK) is a primary growth hormone that fine-tunes plant development under normal and stressful conditions. Floral induction and parthenocarpy in horticulture crops significantly decrease the duration of the transition period and the amount of work needed for pollination. In the case of protected horticulture adopted mostly for *Cucurbitaceae* and *Solanaceae*, temperature and humidity often fluctuate, affecting the overall growth and yield. CK application in protected horticulture could be beneficial in minimizing the growth and yield penalty. On the other hand, CK provides resistance to horticultural crops against abiotic and biotic stresses. The complex transformation technology in horticultural crops made it a hectic job to study the molecular role of CK biosynthesis and signaling genes in growth and stress biology. Despite the plethora of literature, we discussed here, gaps and loopholes are at large and would need to be addressed in future research. Below given are some unsolved puzzles that need attention.

CK is potent during the induction of femaleness, as reported in kiwi. Ethylene and auxin have been previously reported to induce femaleness in horticultural crops [37]. Therefore, it is vital to understand whether CK-mediated femaleness depends on the other two actors (auxin and ethylene).

The salt tolerance mechanism of CK is perplexing and requires additional investigation. The CK crosstalk with other hormones could be of particular interest and requires investigation.

Despite the promising research findings, the use of CK in combating abiotic stresses such as heat, cold, waterlogging, and heavy metals has been overlooked. For instance, heat stress often occurs in protected horticulture environments, particularly on hot and sunny days. It will be interesting to study the role of exogenous CK in crops such as tomato and cucumber subjected to heat stress,

CK application ceded positive results against sucking insect pests in rice [158]. Aphids pose a substantial challenge in protected horticulture, resulting in extensive harm to plant development and productivity. CK is a viable option to examine since it has the potential to reduce the negative impact of aphids and other sucking pests.

Viruses greatly affect the productivity of horticultural crops. The exogenous CK significantly suppresses the virus pathogenicity, yet it is widely neglected in the field of horticultural plant virology.

Genetic engineering techniques such as CRISPR Cas9 could be used to study the role of CK biosynthesis and signaling genes under abiotic and biotic stresses.

## Data Availability

All the data generated in this study are included in the published article.
